# Identification of the Temperature Induced Larvicidal Efficacy of *Agave angustifolia* against *Aedes, Culex*, and *Anopheles* Larvae

**DOI:** 10.3389/fpubh.2015.00286

**Published:** 2016-01-12

**Authors:** Mithilesh Kajla, Kurchi Bhattacharya, Kuldeep Gupta, Ujjwal Banerjee, Parik Kakani, Lalita Gupta, Sanjeev Kumar

**Affiliations:** ^1^Vector Biology Laboratory, Department of Biological Sciences, Birla Institute of Technology and Science, Pilani, India; ^2^Graduate School of Biological Sciences, University of Cologne, Cologne, Germany; ^3^Institute of Genetics and Molecular and Cellular Biology, Illkirch, France

**Keywords:** mosquito, vector-borne diseases, larvicidal activity, plant extract, *Agave angustifolia*

## Abstract

Synthetic insecticides are generally employed to control the mosquito population. However, their injudicious over usage and non-biodegradability are associated with many adverse effects on the environment and mosquitoes. The application of environment-friendly mosquitocidals might be an alternate to overcome these issues. In this study, we found that organic or aqueous extracts of *Agave angustifolia* leaves exhibited a strong larvicidal activity (LD_50_ 28.27 μg/ml) against *Aedes aegypti*, *Culex quinquefasciatus*, and *Anopheles stephensi* larvae within a short exposure of 12 h. The larvicidal activity of *A. angustifolia* is inherited and independent of the plants vegetative growth. Interestingly, the plant larvicidal activity was observed exclusively during the summer season (April–August, when outside temperature is between 30 and 50°C) and it was significantly reduced during winter season (December–February, when the outside temperature falls to ~4°C or lower). Thus, we hypothesized that the larvicidal components of *A. angustifolia* might be induced by the manipulation of environmental temperature and should be resistant to the hot conditions. We found that the larvicidal activity of *A. angustifolia* was induced when plants were maintained at 37°C in a semi-natural environment against the controls that were growing outside in cold weather. Pre-incubation of *A. angustifolia* extract at 100°C for 1 h killed 60% larvae in 12 h, which gradually increased to 100% mortality after 24 h. In addition, the dry powder formulation of *A. angustifolia*, also displayed a strong larvicidal activity after a long shelf life. Together, these findings revealed that *A. angustifolia* is an excellent source of temperature induced bioactive metabolites that may assist the preparedness for vector control programs competently.

## Introduction

Mosquitoes are infamous vectors for numerous life-threatening diseases. Synthetic chemicals (insecticides) are mostly employed to control the vector population. However, the disadvantages associated with their applications warrant the discovery of environment-friendly approaches to control mosquitoes at various stages of their development.

Mosquito developmental stages include both aquatic and terrestrial life. Aquatic life starts after the female lays eggs in moist conditions. They further develop into four different stages of instar larvae and then into pupae. Pupa finally turns into a flying adult. The relatively long, ~8–10 days, aquatic cycle of mosquito larvae development is considered a potent target for controlling its population. Synthetic larvicidals are mainly employed to achieve this goal (1, 2). The use of non-biodegradable larvicidals is also paralleled by major drawbacks such as killing of beneficial organisms and biological accumulation through the food chain that resulted in numerous deleterious effects on ecological systems (3). In addition, the poor human acceptance of insecticide spray and development of insecticide-resistant mosquitoes are also major threats in this area (4).

In the context of above facts, secondary metabolites from the botanical world (called natural phyto-larvicides) may also be employed for controlling mosquito population. These metabolites are easily obtained at reasonable cost and their intrinsic biodegradable nature makes them the best suitable for this purpose (5). The crude extracts of several plants in numerous polar/non-polar solvents, such as water, hexane, methanol, chloroform, ethanol, and acetone, are reported to exhibit larvicidal activity (5–8). However, there are certain limitations in their preparation, stability, and efficacy against different mosquitoes genera. These facts warrant the discovery of novel phyto-larvicides that can efficiently control the population of major disease vectors and will certainly help in reducing the spread of numerous deadly infections among humans.

We screened several randomly selected plants to study their larvicidal properties. Interestingly, one of the plants *Agave angustifolia* “Marginata” also known as Caribbean *Agave* revealed a potent mosquito larvicidal activity. *A. angustifolia* (family *Agavaceae*) is a medium-sized monocotyledonous plant with a dense round rosette atop a very short trunk. This xerophytic plant is a robust survivor and tolerant to hot and dry environments (9). The genus *Agave* has more than 275 species that are globally distributed and *A. angustifolia* is a common weed or sometime used as ornamental plant in our region. This plant mostly propagates through vegetative reproduction, either by rhizomes or by bulbils, and forms aggregations of individual plants. However, the sexual reproduction is also reported in this plant (10, 11). In fact, *Agaves* are of economic importance as sources of fiber, steroids, spirits, and other useful products (12).

The aim of this study was to evaluate the larvicidal nature of *A. angustifolia* leaf extracts against three major human disease vectors, namely *Aedes aegypti, Culex quinquefasciatus*, and *Anopheles stephensi* and understanding the novel features of this plant to establish its applicability for controlling mosquito population at grass-root level.

## Materials and Methods

### Rearing of Mosquito Larvae in the Lab Conditions

*Aedes aegypti*, *C. quinquefasciatus*, and *A. stephensi* larvae were reared in an insectory at 28°C and 80% relative humidity with a photoperiod of 12 h light/dark cycles following all other standard rearing conditions (8, 13). In brief, the mosquito larvae were maintained in plastic trays and fed on a 1:1 mixture of dog food (PetLover’s crunch milk biscuit, India) and fish food (Gold Tokyo, India). Adults were regularly maintained at 10% sucrose solution *ad libitum*. For colony propagation, 3-4 days old females were starved for 24 h and fed on anesthetized mice. These mice were maintained in a pathogen-free environment inside the animal facility and all the procedures were followed in accordance with the research policies that were approved by the organization’s animal ethics committee. The eggs laid by these females in moist conditions were collected and the hatched larvae were floated in the water to continue the cycle.

### Larvicidal Activity of *Agave* Leaf Extracts

**Figure 1:**
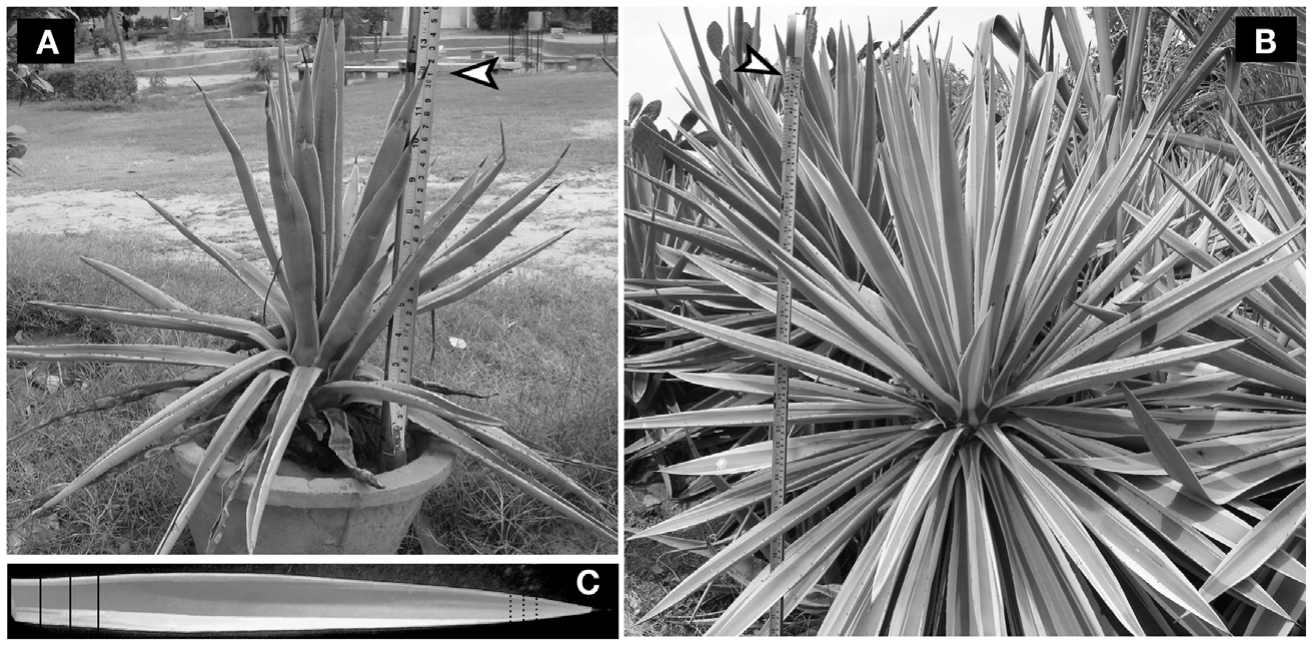
**Representative *Agave angustifolia* plants selected for larvicidal activity**. *Agave angustifolia* plants with different heights, which denote their vegetative growth, were selected for analyzing the larvicidal activity in their leaf extracts. **(A)** Smaller size plants (12″ in height) growing in a pot or **(B)** a larger size plants (44″ in height) growing in an open field are shown here. **(C)** *Agave angustifolia* leaf depicting the pattern of slicing for crude extract preparation. Solid and dotted vertical lines indicate leaf areas proximal or distal to the stem, respectively. Black arrowheads indicate the height of each plant on an inch scale.

*Agave angustifolia* plants growing in the University campus, roadsides in the nearby locations (geographical coordinates: 28° 22′ 0″ North, 75° 36′ 0″ East) or potted were used in this study (representative plants are shown in Figures 1A,B). This plant is a wild weed and, therefore, no permission was obtained for their usage. *A. angustifolia* leaves, freshly excised from the plants, were used to prepare extracts in different organic solvents, such as hexane, acetone, ethanol, or aqueous solvents as before (6). Briefly, the leaves were washed in water, sliced as shows in Figure 1C, and their flesh was finely triturated with the help of a mortal pestle to prepare a thick paste. Fifteen grams of the paste were transferred to a 50 ml centrifuge tube and 30 ml solvent, either acetone, ethanol, hexane, or water was added to the paste and mixed properly. The tube was rocked for 2 h at room temperature (RT), centrifuged at 2300 × *g* for 10 min and debris-free supernatant was collected in a fresh tube.

Twenty five to forty 4^th^ instar larvae of *A. aegypti*, *C. quinquefasciatus*, or *A. stephensi* were allowed to float in water cups supplemented with various doses (20, 50, 100, 200 μg/ml) of the leaves extracts that were prepared in either of the solvent. The solvent alone was added in corresponding amounts to the sham-treated controls. Percentage mortality of the larvae was calculated in each treatment against their respective controls at the stipulated time (12, 24, and 36 h) and represented as mean ± SD. The LD_50_ value was calculated by the probit analysis method as before (13). Quantitative measurement of the crude extract was carried after evaporating the solvents in speed vac. Each experiment was performed in triplicates and repeated at least thrice to confirm the findings. The statistical significance of the data (where *p* is <0.05) was confirmed by *t*-test (we verified our data for normal distribution before performing *t*-test) and one-way ANOVA. The sigma plot (SigmaPlot 10.0 Systat Software, San Jose, CA, USA) and sigma STAT plus software (StatPlus v5, AnalystSoft Inc., statistical analysis program) were used to prepare graphs and performing the statistical analysis of data, respectively.

### Effect of Environmental Conditions on the Regulation of Larvicidal Activity in *Agave* Plants

To understand the effect of environmental temperature on induction of larvicidal activity in *A. angustifolia*, we compared the larvicidal activity in plants those were maintained at different temperatures in semi-natural environments. For that, individually potted *A. angustifolia* plants were kept at either 37 or 4°C in plant growth chambers. Except the temperature, all other natural environmental conditions (light intensity ~1000 lux, humidity 30–50% as present in the open environment at the time of study) for these plants were maintained same. After 2 days of incubation, the larvicidal activities in their leaf extracts were determined. The plants with similar vegetative growth and growing outside either in cold or hot weather, respectively, served as controls for these experiments. Percentage mortality of larvae and statistical significance of the data were calculated as above.

### Larvicidal Activity in *Agave* Dry Leaf Powder

For preparation of the dry powder, we took the fresh leaves and determined the larvicidal activity in their aqueous extract as mentioned above. *A. angustifolia* leaves that exhibited strong larvicidal activity in their aqueous extracts were subjected to sun-, oven-, or shade-drying process. The dried leaves were grinded in a mixer and stored as powder in moisture-free conditions. After 3 months of shelf life at RT, 15 g of powder was soaked in 30 ml water for 2 h with continuous rocking. The tube was centrifuged at 2300 × *g* for 10 min and powder-free supernatant was collected in a fresh tube. The powder-free extract or the powder itself, equivalent to the amount of fresh leaves, was analyzed for larvicidal activity as before. Percentage mortality of larvae and statistical significance of the data was calculated as above.

### Analysis of Secondary Metabolites

#### Analysis of Alkaloids

0.5 ml of aqueous extract was mixed with 1.5 ml of 10% acetic acid in ethanol and allowed to stand for 4 h. The mix was filtered and the filtrate was concentrated to one-fourth of the original volume (0.5 ml now) in a water bath at 80°C. Furthermore, 0.025 ml of concentrated ammonium hydroxide was added and the orange color product was read in a spectrophotometer as mentioned above.

#### Analysis of Phenols

0.2 ml of ammonium hydroxide solution and 0.5 ml of amyl alcohol was added to 0.5 ml of plant aqueous extract. The mixture was kept at RT for 30 min to react. The spectra were read for the final greenish-brown colored product as above.

#### Analysis of Steroids

0.5 ml ethanol was added to 0.5 ml aqueous extract and mixed properly. Furthermore, 0.4 ml H_2_SO_4_ and then 0.4 ml of acetic anhydride was added with continuous mixing. The end product with greenish-blue color was read.

#### Analysis of Phlobatannins

0.5 ml aqueous extract was incubated at 80°C with 0.2 ml of 1% aqueous hydrochloric acid for 10 min. The red colored end product was read.

#### Analysis of Flavonoids

0.4 ml of diluted ammonia solution was added to 0.5 ml aqueous extract. After mixing, 0.050 ml concentrated H_2_SO_4_ was added. The end yellowish product was read in spectrophotometer.

#### Analysis of Saponins

Fifteen milliliters of 20% aqueous ethanol was added to 5 g of the leaves paste and incubated at 55°C for 5 h with continuous shaking. After incubation, the mixture was filtered and the residue was re-extracted with 15 ml of 20% ethanol. The combined total filtrate (~30 ml) was reduced to 10 ml in a water bath at 90°C. Five milliliters of diethyl ether were added to the concentrated filtrate and vortexed properly. After centrifugation the aqueous layer was separated and 15 ml of n-butanol was added to it. After vortexing, it was centrifuged and the *n*-butanol layer was collected, washed twice with 5 ml of 5% sodium chloride. The butanol was evaporated in oven and the residual saponins were dissolved in methanol to read in a spectrophotometer.

## Results

### *Agave* Leaf Extracts Proficiently Kill *Aedes, Culex*, and *Anopheles* Larvae

The larvicidal properties of *A. angustifolia* were analyzed against *A. aegypti*, *C. quinquefasciatus*, and *A. stephensi* larvae. For that the crude extracts from the fleshy leaves were prepared in different organic solvents as described for other plants (6–8, 13–16). Since, the polarity of organic solvent is important for the extraction of plant metabolites, we used three different organic solvents, such as hexane, acetone, and ethanol, for this purpose as discussed in Section “Materials and Methods.” We adopted the simplest method of crude extract preparation so that a common user can effortlessly prepare it.

**Figure 2:**
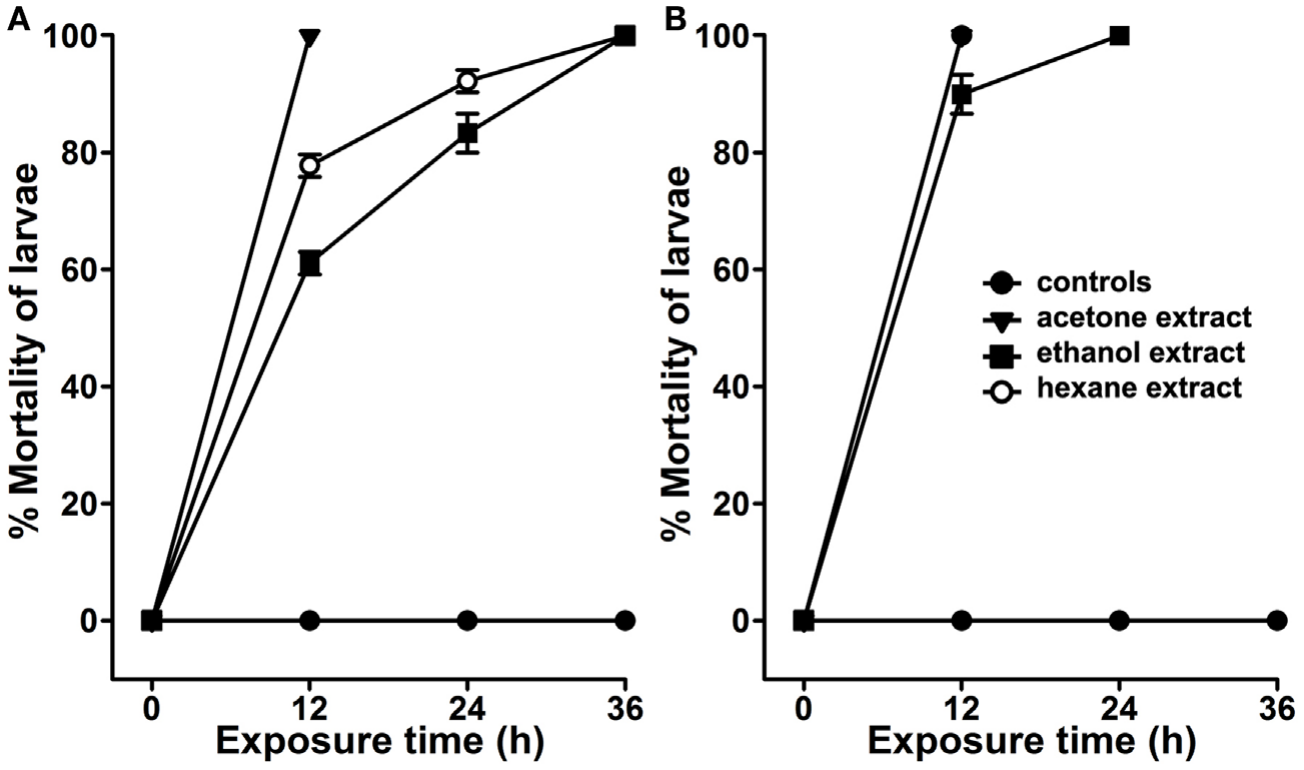
**Larvicidal activity in the organic extracts of *Agave***. The extracts of *Agave angustifolia* leaves were prepared in different organic solvents, such as acetone, ethanol, or hexane. **(A)** *A. aegypti* or **(B)** *C. quinquefasciatus* larvae were treated with 100 ppm dose of these organic extracts separately. The percentage of larval mortality in each extract exposure was calculated against the sham-treated controls and represented as the mean ± SD of triplicates.

We analyzed the larvicidal activity of the organic crude extracts against *A. aegypti* larvae under standard lab-rearing conditions. The larval mortality in controls or those exposed to 100 ppm crude extract are presented in Figure 2A. These results revealed that *A. angustifolia* crude extract in either of the organic solvents is effective to kill *A. aegypti* larvae in a time-dependent manner. The acetone extract is most effective and killed all the larvae within 12 h of exposure. Ethanol extract of *A. angustifolia* demonstrated the same effect at 36 h (100% mortality), however, it killed 61 ± 2% larvae in 12 h. Moreover, the hexane extracts revealed intermediate larvicidal activity and likewise the mortality increased consistently (77 ± 2% to 100%) with time (12–36 h) (Figure 2A).

Several studies reported that organic extracts prepared from other plants exhibit larvicidal activity only against one genus of mosquito (6–8, 13, 15–17). Thus, to understand the broad larvicidal spectrum of *A. angustifolia* organic extracts, we analyzed their effects against the larvae of other mosquitoes. We found that *A. angustifolia* extract in hexane or acetone also killed 100% *C. quinquefasciatus* larvae within 12 h (Figure 2B). However, the ethanol extract killed 90 ± 3% *C. quinquefasciatus* larvae in the same duration. Interestingly, the larvicidal efficacy of *A. angustifolia* extract in the organic solvents is more pronounced against *C. quinquefasciatus* than *A. aegypti* larvae (Figure 2). In addition, we found that the above-mentioned *A. angustifolia* organic extracts also have uniform lethality against *A. stephensi* larvae (Figure S1 in Supplementary Material).

**Figure 3:**
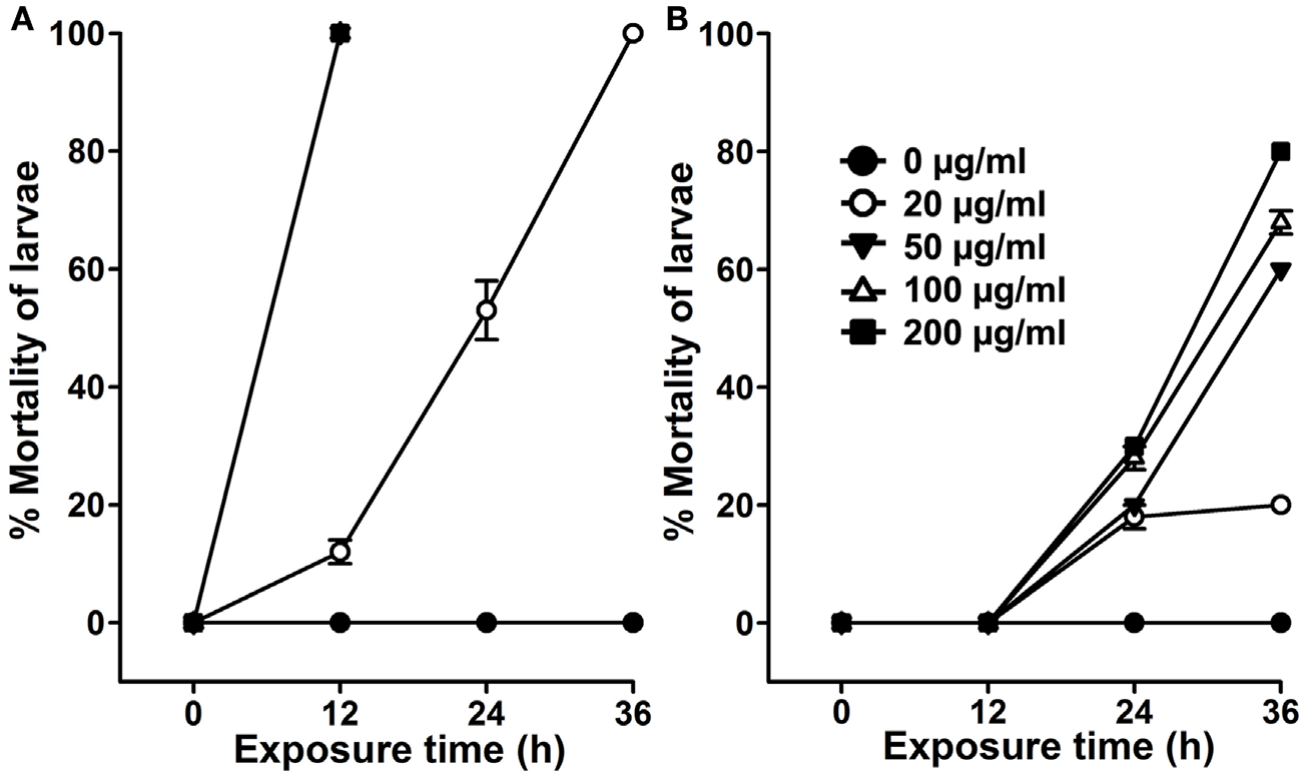
**Effects of seasonal variations on *Agave*-mediated larvicidal activity**. *A. aegypti* larvae were exposed to various doses (0–200 μg/ml) of aqueous extracts prepared from the plant leaves that were growing outside in an open area during **(A)** summer or **(B)** winter season. The percentage mortality of larvae at each time point was calculated against the sham-treated controls (0 μg/ml) and represented as the mean ± SD of triplicates.

**Figure 4:**
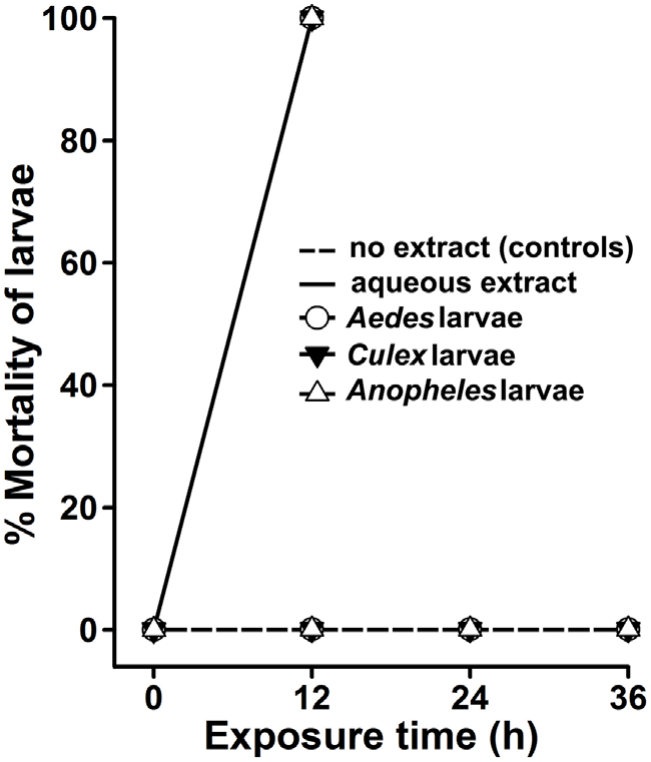
***Agave* aqueous extract effectively kills mosquito larvae**. *A. aegypti*, *C. quinquefasciatus*, or *A. stephensi* larvae were sham treated (no extract, controls) or exposed to 100 μg/ml aqueous extract of *Agave angustifolia*. The percentage mortality of larvae at each time point was calculated against the controls for each genus individually and represented as the mean ± SD of triplicates.

The use of organic solvents in extract preparations may invite some issues, such as their incompatibility and toxicity to the natural environment, cost of preparation, and availability for a common user. Thus, we prepared aqueous extract from *A. angustifolia* leaves and estimated its dose-dependent larvicidal efficacy against *A. aegypti* larvae. Results presented in Figure 3A revealed that 50 μg/ml of *A. angustifolia* aqueous extract is strong enough to kill all the *A. aegypti* larvae within 12 h. The LD_50_ value at 12 h was estimated to be 28.270 μg/ml by the probit analysis method (Table 1). Furthermore, 100 μg/ml *A. angustifolia* aqueous extract also exhibited 100% killing efficiency against *A. aegypti*, *C. quinquefasciatus or A. stephensi* larvae within 12 h (Figure 4). These findings are noteworthy in comparison to other reports where aqueous extract of many plants demonstrated the larvicidal activity, mostly between 24 and 72 h and even later (18–20). This least effectual time for *A. angustifolia*-mediated larval mortality is a unique feature and to the best of our knowledge, it is not reported before.

**Table 1:** **Probit analysis of *Agave angustifolia* larvicidal activity against *Aedes aegypti* larvae**.

Time (h)	N	LD 50 (μg/ml)	Slope ± SE	CL (95%)	Chi-square	*p*-value
12	30	28.270	5.560 ± 0.668	24.183–32.460	0.886	0.642
24	30	19.157	5.016 ± 1.012	14.243–23.001	0.995	0.996

### *Agave* Larvicidal Activity is Highly Thermostable, Inherited, and Independent of Plant Vegetative Growth

**Figure 5:**
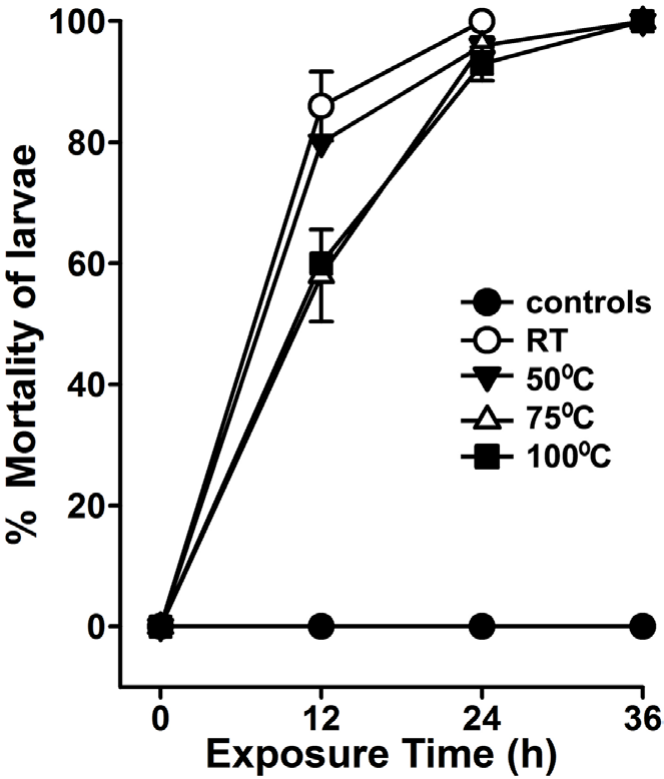
***Agave* aqueous extract-mediated larvicidal activity is highly thermostable**. *A. aegypti* larvae were exposed to 100 μg/ml *Agave angustifolia* aqueous extracts that were pre-incubated at different temperatures for 1 h. Percentage mortality of larvae was calculated at each time point against the sham-treated controls and represented as the mean ± SD of triplicates.

In our region or other parts of the world, the environmental temperature reaches up to 50°C during summer. Because *A. angustifolia* is a drought deciduous plant, thus, we postulated that its larvicidal activity must be mediated by heat-resistant secondary metabolites. To demonstrate that, we pre-incubated the aqueous extract for 1 h at different temperatures before performing the larvicidal assays. Prior incubation of aqueous extract at RT or 50°C killed 86 ± 6 and 80 ± 0% *A. aegypti* larvae, respectively, in 12 h (Figure 5, *p* = 0.2). However, the extracts pre-incubated at 75 or 100°C killed 58 ± 8 and 60 ± 0% larvae, respectively, in 12 h (*p* = 0.007 between RT and 75°C incubated extracts). Importantly, the larvicidal effects of all these pre-incubated extracts are similar at 24 h (Figure 5, *p* = 0.1 between RT and 100°C pre-incubated extract-mediated larval lethality). This heat-resistant larvicidal activity of *A. angustifolia* extract may be useful to control mosquito population in natural warm–hot conditions.

**Figure 6:**
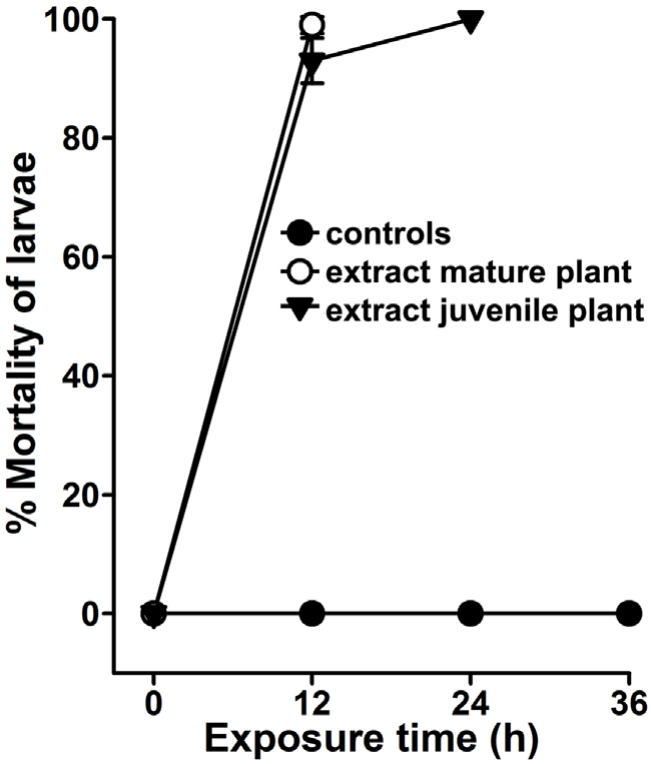
***Agave* larvicidal activity is independent of the plants vegetative growth**. *A. aegypti* larvae were exposed to 100 μg/ml aqueous extract prepared from the leaves of a mature (~44″ in height) or juvenile (~12″ in height) *Agave angustifolia* plants. Percentage larval mortality at each time point was calculated against the sham-treated controls and represented as the mean ± SD of triplicates.

*Agave angustifolia* is generally a medium-sized plant (~48″ in height). Flowering occurs around 10 years of age or much later (9). The plant we selected for larvicidal assays were approximately 44″ in height and, thus, we considered them mature (Figure 1B). To determine the intrinsic larvicidal nature of *A. angustifolia*, we compared the larval lethality in the aqueous extracts of juvenile and mature plants as represented in Figures 1A,B. We found that 100 μg/ml aqueous extract of mature or juvenile plant killed 98 ± 1.4 and 93 ± 4% *A. aegypti* larvae, respectively, at 12 h (Figure 6, *p* = 0.075). On the other hand, we also analyzed the larvicidal activity in other mature and juvenile *A. angustifolia* plants (height ranges from 4 to 50″) collected from different nearby locations and all revealed similar results (Figure S2 in Supplementary Material). This indicates that the larvicidal nature of *A. angustifolia* is not only inherited but also independent of its vegetative growth.

### *Agave* Larvicidal Activity is Induced Exclusively During Summer

We experienced a surprising outcome regarding the larvicidal nature of *A. angustifolia*. Aqueous extracts prepared during summer (April–August, when outside temperature rises up to 45°C or higher) from the plants that were growing in the open field demonstrated a dose-dependent larvicidal activity against *A. aegypti* larvae (Figure 3A; Figure 4). However, the aqueous extract prepared from the same plants revealed reduced and delayed larvicidal activity during winter (December–February, when the outside temperature falls between 4 and 10°C or lower). Results depicted in Figure 3B revealed that 100 μg/ml aqueous extract killed 0 ± 0% *A. aegypti* larvae in 12 h and likewise increased to 68 ± 2% at 36 h of exposure. This larvicidal activity is significantly reduced as well as delayed in comparison to the summer activity (Figures 3A,B). These observations indicated that *A. angustifolia* metabolite(s) mediating larvicidal activity in aqueous extract is/are induced by the environmental conditions, at least the higher temperature. This may be due to the specific physiological role of these metabolite(s) under given circumstances.

### Manipulation of Environmental Temperature Modulates *Agave* Larvicidal Activity and Profiling of Secondary Metabolites

**Figure 7:**
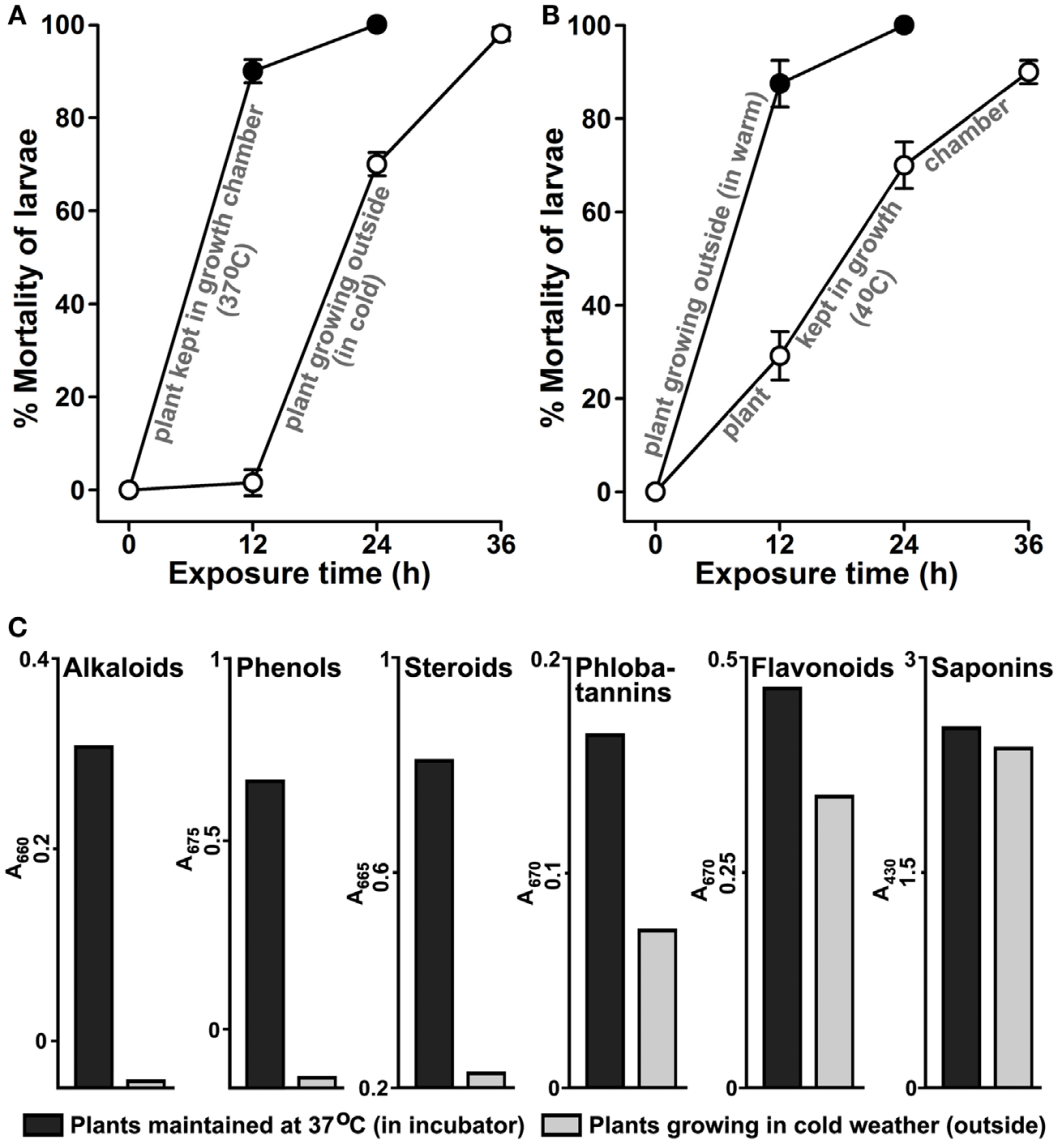
**Manipulation of environmental temperature induces *Agave*-mediated larvicidal activity**. The effect of environmental temperature on the induction of larvicidal activity in *Agave angustifolia* was analyzed against *A. aegypti* larvae. For that, the larvicidal activity in 100 μg/ml aqueous extract was compared from the plants that were growing or maintained in two different environmental conditions. The comparative larvicidal activities are shown here in those plants that were growing **(A**) outside in the cold environment during winter season and similar plants maintained at 37°C in a plant growth chamber or **(B)** outside in hot environment during the summer season and similar plants maintained at 4°C in a growth chamber as discussed in Section “Material and Methods.” The percentage mortality of larvae at each time point was calculated against the sham-treated controls (not shown here) and represented as the mean ± SD of triplicates. **(C)** The relative levels of various secondary metabolites were compared in the aqueous extracts of *Agave angustifolia* plants maintained at 37°C in a plant growth chamber or growing in external environment during cold season as mentioned in the panel A. The values represent absorption maxima **(A)** in visible range for each metabolite separately.

Our results indicated that environmental conditions seem to be influencing the larvicidal properties of *A. angustifolia* (Figure 3). We hypothesized that during winter if *A. angustifolia* plants are maintained under warm conditions, their larvicidal activity may be induced. We tested this fact after swapping juvenile plants ~12″ in height (Figure 1A) growing in individual pots from outside cold winter environment to 37°C in a plant growth chamber and compared their larvicidal activity to those growing outside. The aqueous extract from the plants maintained under warmer conditions revealed stronger lethality against *A. aegypti* larvae than the plants growing outside in winter (Figure 7A). Aqueous extract prepared from the plants that were maintained in a warm incubator killed 90 ± 3% larvae at 12 h of exposure. However, the similar control plant growing outside in the cold weather killed 1.6 ± 2.8% larvae in same time (*p* = < 0.001). In addition, we also performed the similar experiment during summer and shifted the plants from outside hot to 4°C, keeping rest conditions similar to the natural environment, and performed larvicidal assays. The results illustrated in Figure 7B revealed that the plants growing outside in hot summer conditions killed 87 ± 5% and those maintained at lower temperature killed 29 ± 5% *A. aegypti* larvae at 12 h (*p* = < 0.001). This indicated that, at least, one of the abiotic stress factors (i.e., increased temperature) stimulates the production of those secondary metabolite(s) in *A. angustifolia* that exhibit(s) larvicidal activity. Overall, these results demonstrated that the *A. angustifolia* is equipped with active larvicidal metabolites mostly during summer; however, the semi-natural warmer environment under the laboratory conditions can also replicate the same effect.

Our results confirmed that the larvicidal activity in *A. angustifolia* is induced at least by environmental temperature (Figures 7A,B). Thus, we hypothesized that abiotic stress, i.e., temperature may alter the composition of metabolites and due to the production of specific compound(s) *A. angustifolia* exhibits distinct larvicidal activity. To demonstrate this, we analyzed and compared the levels of various secondary metabolites in those plants that are discussed in Figure 7A. The plant maintained in warm environment has elevated levels of several secondary metabolites, such as flavonoids, phenols, alkaloids, phlobatannins, and steroids in comparison to the plant growing at a low temperature (Figure 7C). In addition, when we analyzed these metabolites in plants as mentioned in Figure 7B, their levels also revealed similar patterns (Figure S3 in Supplementary Material). These findings correlated an association between the external environment-induced production of secondary metabolites and larvicidal activity in *A. angustifolia* aqueous extracts.

### *Agave* Dry Leaf Powder Formulation Also Exhibits Strong Larvicidal Activity

**Figure 8:**
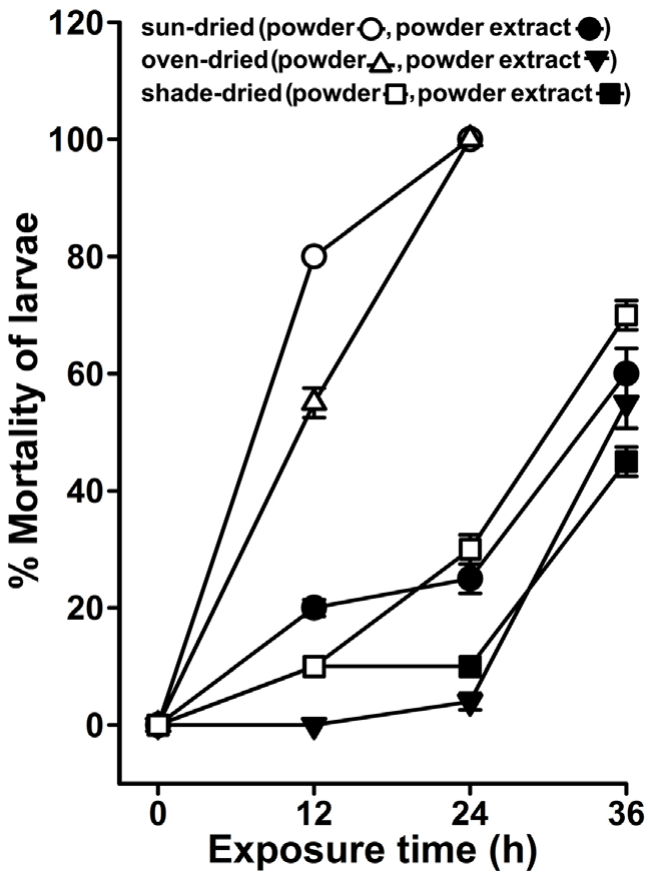
**The larvicidal activity of *Agave* dried powder formulation**. *Agave angustifolia* leaves with active larvicidal activity were either sun-, oven-, or shed-dried and the powder was prepared. The powder was stored at room temperature under moisture-free conditions for 3 months (the shelf life of the powder). *A. aegypti* larvae were exposed to the powder-free aqueous extracts or powder directly added to the water in the amount equivalent to the fresh leaves exhibiting the larvicidal activity as mentioned in Figure 4. The percentage mortality of the larvae at each time point was calculated against sham-treated controls (not shown here) and represented as the mean ± SD of triplicates.

The larvicidal activity of *A. angustifolia* is although inherited, but appears to be prominent exclusively during summer (Figure 3). This limited duration of available *A. angustifolia* larvicidal activity in natural conditions impedes its utilization throughout the year. To overcome these limitations, we assumed that the leaf with larvicidal activity (Figure 4) could be dried and stored as powder for future usage. We prepared the powder from sun-, oven-, or shade-dried leaves and after 3 months of shelf life, the larvicidal activity was analyzed in the powder or powder-free extract against *A. aegypti* larvae as mentioned in Section “Materials and Methods.” We found that the powder-free extract from sun-, oven-, or shade-dried leaves exhibited 20 ± 1.4, 0 ± 0, and 10 ± 0% larval mortality, respectively, after 12 h (Figure 8), which was remarkably less in comparison to the original leaf extract-mediated anti-larval activity (Figure 4). Furthermore, these extracts could increase the larval mortality to 60 ± 4, 55 ± 4, and 45 ± 2.5, respectively, at 36 h (Figure 8). Interestingly, the sun- and oven-dried leaf powder itself killed 80 ± 0 and 55 ± 3.5% larvae, respectively, in 12 h that increased gradually to 100% mortality at 24 h (Figure 8). However, the powder prepared from shade-dried leaves revealed minimum mortality (10 ± 0 to 70 ± 2.5%) with time (12–36 h). In conclusion, dried leaf powders have slightly delayed larvicidal activity against the original leaf extract. Interestingly, the dried leaf powder-mediated larval lethality is dependent on the method of drying the original leaf and sun-dried leaf powder could reveal stronger larvicidal effects among others (Figure 8). The inability of powder-free aqueous extract to kill larvae may be due to inadequate extraction of active larvicidal compounds from the powder due to the limitations of our extraction methods. Furthermore, the larvicidal activity in powders itself may be due to time-dependent leaching of larvicidal components into the water and that may explain those slightly delayed killing effects (Figure 8).

## Discussion

Crude extracts prepared from the whole plant or specific parts of the plant, such as leaf, stem, fruit, and root, have been reported to exhibit potent lethality against insect larvae (5, 6, 15) and, hence, we termed them phyto-insecticides. These phyto-insecticides are biodegradable and reduce the environmental chemical burden as posed by the synthetic chemicals. In this study, we found that the crude extract prepared from *A. angustifolia* by a simple, least labor-intensive and cost-effective method exhibits strong larvicidal activity against *A. aegypti*, *C. quinquefasciatus*, and *A. stephensi* larvae within 12 h (Figure 4). This is the minimum time reported for a plant crude extract to kill all the larvae of medically important mosquitoes and upholds a promising future to control vector population.

Larvicidal properties of natural compounds vary from one plant to another and may have a low degree of effectiveness only against selected genera of mosquitoes. The variations in the larvicidal efficacy of plant extracts are highly dependent on the type or the polarity of the solvent employed for extraction purpose (5). Therefore, the solvents with varying degree of polarity, such as hexane, petroleum ether, methanol, benzene, ethyl acetate, methanol, chloroform, and acetone, are generally used for this purpose (5, 15, 16, 21). The use of unrelated solvents for the preparation of plant crude extracts sometimes reveals that the extraction efficiency of one solvent is superior over the other solvents (16, 18). Surprisingly, we found that the crude extracts of *A. angustifolia* in individual solvents of varying polarity (ethanol and hexane) have more pronounced larvicidal effect against *C. quinquefasciatus* than *A. aegypti* larvae (Figure 2). These findings support the previous studies where lethal doses of *Eichhornia crassipes* and *Artemisia nilagirica* plant extracts vary for two different genera of the mosquitoes (7, 8). This may be due to the presence of diverged bioactive compounds in *Agave* or the varying solubility of the same compound in different solvents. In this condition, preparing the plant extract in a mixture of two or more solvents may enhance its mosquitocidal activity (16, 18). In our experiments when we employed similar strategies to prepare *A. angustifolia* extract by using a mixture of solvents, we did not find any additional effect on its larvicidal properties (Figure S4 in Supplementary Material). This simply may be due to the limitations of our extraction methodology.

In general, the organic solvents used for plant extraction might have a concern for “cost of preparation” and may end up in escalating the chemical burden or toxicity in the natural environment as well. Their incompatibility to the aqueous environment is also an associated concern. Therefore, we planned to analyze the larvicidal activity in a simply prepared aqueous extract of *A. angustifolia*, which satisfies all the concerns related to organic solvent extractions. Interestingly, the aqueous extract of *A. angustifolia* also exhibited a strong larvicidal activity against *A. aegypti*, *C. quinquefasciatus*, and *A. stephensi* larvae (Figure 4). This is in contrast to some other reports where organic extracts from plants exhibit strong larvicidal activity; however, their aqueous extracts remain inactive (22).

While planning strategies to apply plant extracts for controlling the larval population especially in warm countries, it may be worthwhile to identify those biolarvicides that exclusively withstand the environmental temperature. In other words, the discovery of temperature-resistant biolarvicides may be helpful in this direction. Although many of the plants have larvicidal activity in their aqueous extracts; however, this activity is lost with time and/or increased temperature (19, 23). On the contrary, we found that *A. angustifolia* aqueous extract-mediated larvicidal activity was mostly retained in the crude extract after heat treatment (Figure 5), which may signifies its advanced usage in controlling mosquito populations in the natural environment of tropical countries. These findings also highlight that it will be important to investigate the mechanism by which *A. angustifolia* acquires heat-resistant larvicidal activity.

The world of plants is unique in terms of producing different compounds or metabolites that may be directly linked to their stages of vegetative development, environmental conditions, or geographical locations (24). Previous reports suggested that the larvicidal efficacy of phytochemicals varies with plant age; young tissues have greater larvicidal activity than the older ones or sometimes may be *vice versa* (5). Thus, a consistency of larvicidal activity in the plant is an important concern in this field. We analyzed different sized *A. angustifolia* plants, which denote their levels of maturity and vegetative growth, and found that the larvicidal activity is exhibited by all of them (Figure 6 and Figure S2 in Supplementary Material). These findings clearly indicated that the larvicidal nature of *A. angustifolia* is an inherited feature and that is independent of its vegetative growth. These findings are not in agreement with the other reports where only the mature plants exhibited the larvicidal activity (5). In addition, geographical location of the plant also seems to be affecting its larvicidal properties. In case of *Annona squamosa*, the plant leaves collected from different eco-zones possess various compositions of secondary metabolites and exhibit differential larvicidal activity (24). However, when we collected *A. angustifolia* samples from different nearby areas, they resulted in similar larvicidal activity against mosquito larvae (Figure S2 in Supplementary Material).

In addition, some published reports indicated that another species *Agave sisalana* also exhibited positive larvicidal activity (25, 26). In the first study, waste residues after sisal fiber separation from *A. sisalana* leaves were found to be effective in killing *A. aegypti* or *C. quinquefasciatus* larvae within 24 h. However, the effective lethal dose of these fiber residues against *A. aegypti* larvae was almost twice to that of *C. quinquefasciatus* larvae (25). In the second study, the liquid waste of *A. sisalana* killed 100% *A. aegypti* larvae within 24 h at 20 mg/ml concentration and the LC_50_ value was found to be 5.9 mg/ml (26). Interestingly, a study was performed directly from the *A. sisalana* leaves extracts and found that 2% dilution of the extract revealed 100% mortality against *A. stephensi* larvae, however, 1% dilution exhibited same results in case of *C. quinquefasciatus* and *A. aegypti* larvae (11). It is also noteworthy to mention that the leaf extract prepared from one more species of *Agave*, *Agave americana* also revealed 100% mortality against fourth instar larvae of *Anopheles*, *Aedes*, and *Culex* mosquitoes at a concentration of 0.08% within 24–48 h (27). In nutshell, all these studies indicated that although other species of *Agave* also exhibit larvicidal activity, however, their larvicidal efficacy is variable against different mosquito larvae. Interestingly, our study revealed that a single dose (100 μg/ml or 0.01%) of *A. angustifolia* extract killed *Aedes, Culex*, and *Anopheles* larvae in a short duration of 12 h (Figure 4).

The environmental conditions sometimes have a direct effect on plant physiology and behavior. Plants frequently encounter adverse abiotic conditions, such as salinity, drought, freezing, and elevated environmental temperatures. Stress responses in plants are dynamic and engage complex crosstalk at different regulatory levels. Plants might overcome these stresses through avoidance or tolerance, which includes metabolic adjustment through alteration of compatible solutes or secondary metabolites (28, 29). Our observations regarding the induction of *A. angustifolia* larvicidal activity, exclusively during summer, might be an example of environmental abiotic stress. We believe that the plant might be producing some specific metabolites to counteract those stresses and some of these metabolites are also mediating larval lethality (Figures 4 and 7).

Variations in the temperature, an abiotic environmental factor, also alter the composition of plant metabolites. Studies showed that the plant *Panax quinquefolius* growing just at the difference of +5°C contains a higher concentration of storage root ginsenosides than the ones growing at lower temperature (30). Metabolic profiling of *Arabidopsis* indicated that 143 and 311 metabolites were altered in response to heat and cold shocks, respectively. Interestingly, the comparison of these heat- and cold-shock responses revealed that the majority of heat-shock responses were shared with cold-shock responses (31). However, in case of *A. angustifolia*, this may not be true as the metabolites exhibiting strong larvicidal activity seems to be induced exclusively during summer but not in the winter (Figure 3).

Our study revealed that the levels of flavonoids, phenols, alkaloids, phlobatannins, and steroids are higher in those *A. angustifolia* plants that were maintained in warmer environments (Figure 7C and Figure S3 in Supplementary Material). This profiling of secondary metabolites correlated the observed larvicidal activity in these plants (Figures 7A,B). These results are in agreement with other studies where several compounds, such as terpenes, flavones, xanthones, steroids, resins, flavonoids, alkaloids, anthroquinones, anthocyanins, terpenoids, glycosides, phenols, and saponins are mostly responsible for mosquitocidal activities either individually or in combinations (5, 13, 20, 32). In our study, we did not find any significant difference in the levels of saponins in these plants (Figure 7C and Figure S3 in Supplementary Material). Further studies are required to isolate and identify the active principles involved in *A. angustifolia-*mediated larvicidal activity and their mode of action. Our group is actively engaged in that direction.

Larvicidal activity in *A. angustifolia* is not perquisite for all seasons (Figure 3). However, the plant leaves with larvicidal activity may be stored in the form of dehydrated powder to avoid the fermentation of its active components, easy transportation, and for future applications. Our study found that *A. angustifolia* powder-free aqueous extract exhibited low larvicidal activity in comparison to the powder itself, which may be due to inadequate extraction of larvicidal metabolites in our preparations (Figure 8). In addition, powder-mediated larvicidal activity is highly dependent on the process of drying the wet leaves. This may be due to the quantitative/qualitative alteration of the active larvicidal metabolites during drying-mediated osmotic stresses as reported in other plants where air- or sun-drying markedly affected the levels of both primary and secondary metabolites (33, 34). We look forward for *A. angustifolia* dry powder formulated preparations to enhance its potency and stability with minimal adverse effects on the environment. This could help to design efficient strategies for *A. angustifolia* extract-mediated mosquito controls.

In conclusion, the plants displaying larvicidal activity portray a noteworthy attention over the synthetic chemicals due to their biodegradable nature. *A. angustifolia* aqueous extract that was prepared by the least labor-intensive and cost-effective method display a strong larvicidal activity against three major human disease vectors *A. aegypti*, *C. quinquefasciatus*, and *A. stephensi* within a short exposure. Interestingly, the larvicidal activity of *A. angustifolia* is heat-resistant and induced under the defined conditions, therefore, it may be easily applicable at grass-root levels to control mosquito population.

## Author Contributions

MK and KB equally contributed to this work. MK carried and analyzed thermostability, metabolic profiling, and seasonal variations study. KB initiated the work, standardized the protocols, and analyzed the activity and dose kinetics of various extracts against different larvae. KG, UB, and PK established plant maturity and activity relationship and helped in seasonal variations studies. LG, PK, and SK performed dry powder formulation studies, analyzed the data, and wrote the manuscript with input from all authors. All authors read and approved the manuscript.

## Conflict of Interest Statement

The authors declare that the research was conducted in the absence of any commercial or financial relationships that could be construed as a potential conflict of interest.
