# Supplementary material for: Identification of the Temperature Induced Larvicidal Efficacy of Agave angustifolia against Aedes, Culex, and Anopheles Larvae
Source: Front Public Health. 2016 Jan 12;3:286. doi: 10.3389/fpubh.2015.00286 (PMC4709459; doi:10.3389/fpubh.2015.00286)
Supplement: Supplementary file 1 [file Data_Sheet_1.PDF]

frontiers

Supplementary Material

Identification of the temperature induced larvicidal efficacy of *Agave angustifolia* against *Aedes, Culex* and *Anopheles* larvae

Mithilesh Kajla[#], Kurchi Bhattacharya[#], Kuldeep Gupta, Ujjwal Banerjee, Parik Kakani, Lalita Gupta, Sanjeev Kumar*

[#]These authors contributed equally

*** Correspondence:** Sanjeev Kumar: sanjeev@pilani.bits-pilani.ac.in

1 Supplementary Figures

1.1 Supplementary Figure 1

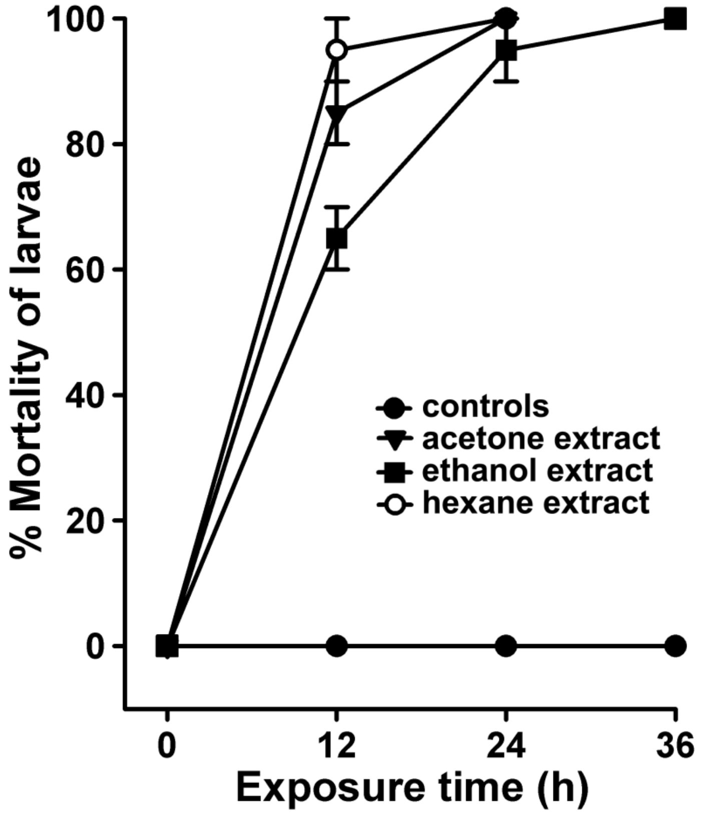

Figure S1: Larvicidal activity in the organic extracts of *Agave* against *A. stephensi* larvae. The extracts of *Agave angustifolia* leaves were prepared in different organic solvents such as acetone, ethanol and hexane. *A. stephensi* larvae were treated with 100 ppm dose of these organic extracts separately. The percentage of larval mortality in each exposure was calculated against the sham treated controls and represented as the mean ± SD of triplicates.

1.2 Supplementary Figure 2

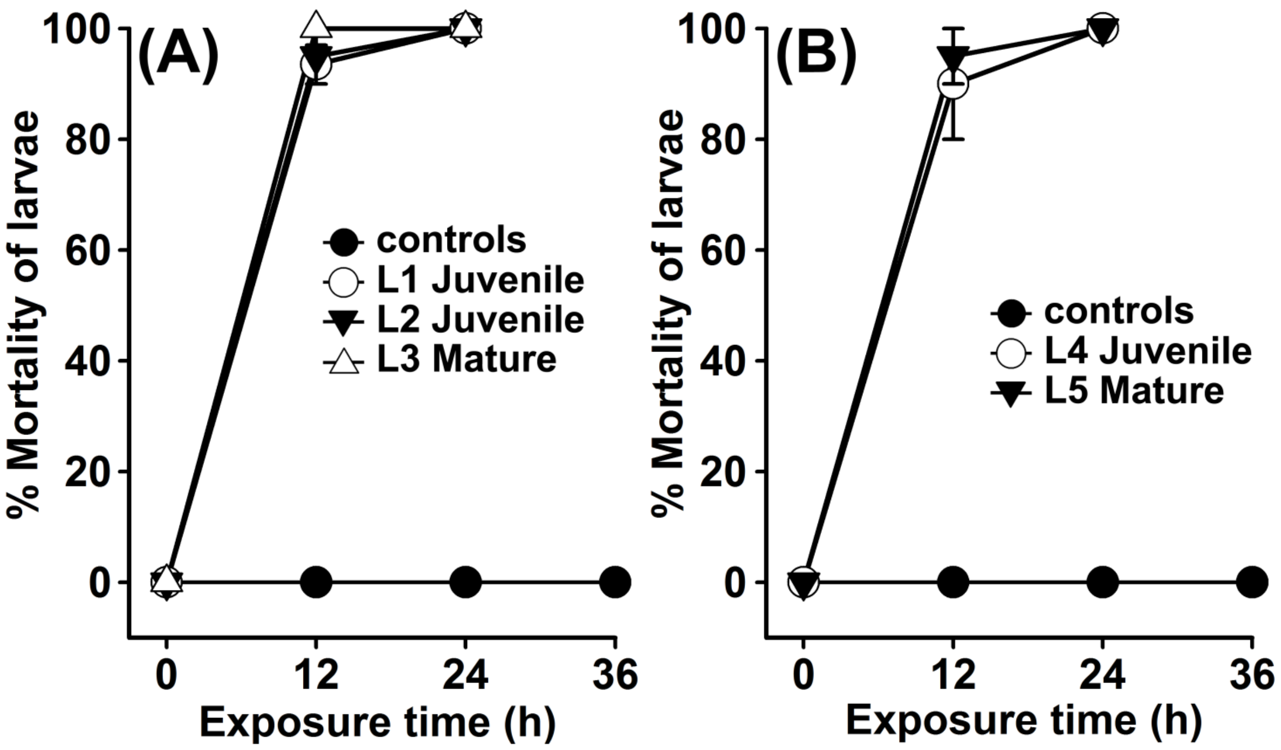

Figure S2: Larvicidal activity of *Agave* collected from different locations. Aqueous extracts were prepared from the leaves of mature and juvenile *Agave angustifolia* plants that were collected from five different locations (L) within the 400 hectare area of the university campus and nearby roadsides. **(A)** *A. aegypti* or (**B**) *A. stephensi* larvae were exposed to 100 µg/ml dose of each extract separately. Percentage larval mortality at each time point was calculated against the sham treated controls and represented as the mean ± SD of triplicates.

1.3 Supplementary Figure 3

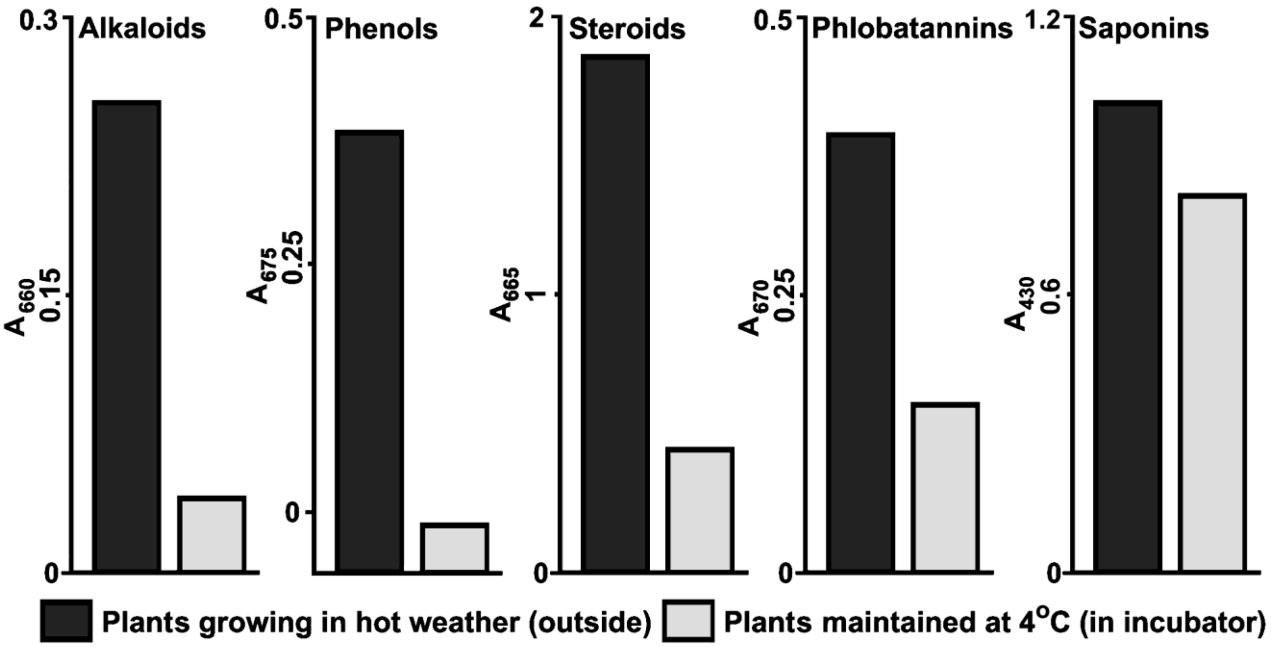

Figure S3: Secondary metabolite profiling of *Agave*. The relative levels of various secondary metabolites were compared in the aqueous extracts of identical *Agave angustifolia* plants maintained either at 4°C in a plant growth chamber or growing in the external environment during summer season as mentioned in Materials and Methods. The values represent absorption maxima (A) in visible range for each metabolite separately.

1.4 Supplementary Figure 4

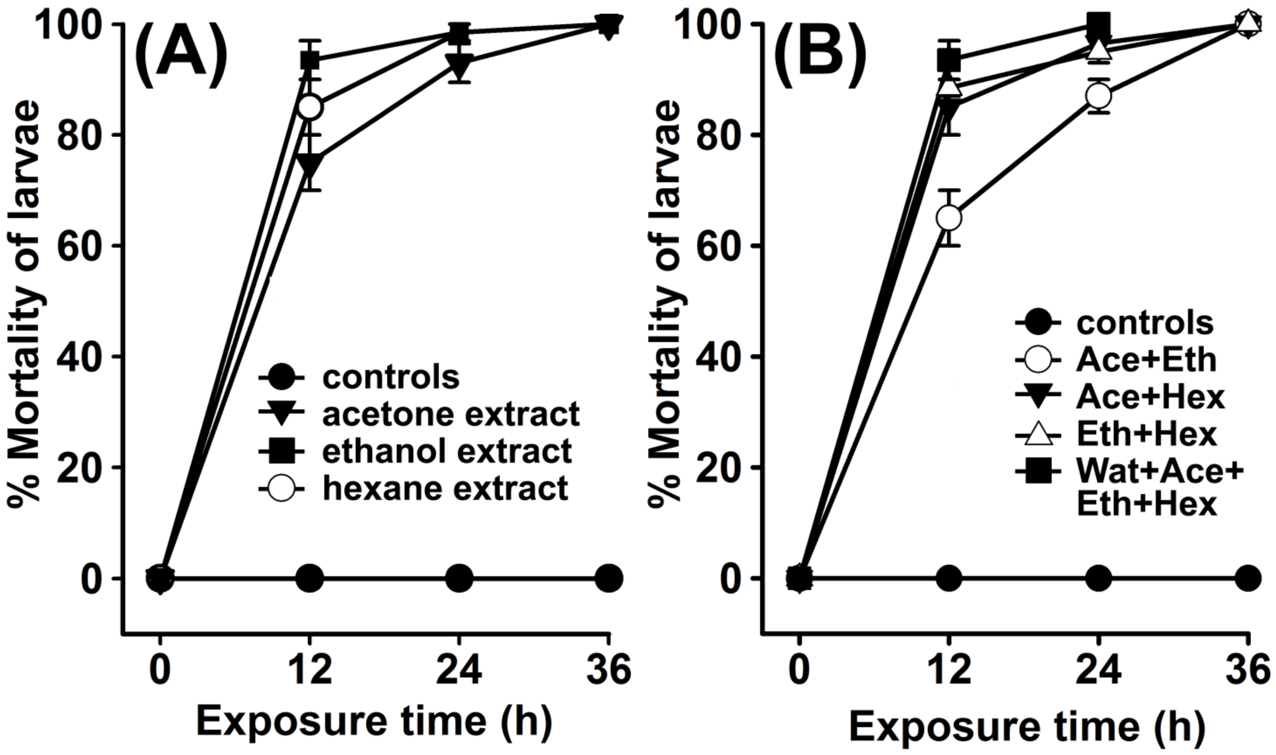

Figure S4: Larvicidal activity of *Agave* extract in the combinations of different organic solvents. The extracts of *Agave angustifolia* leaves were prepared either in **(A)** one particular organic solvent such as acetone, ethanol or hexane or (**B**) in the combination of two or more solvents in equal ratio as depicted in the figure**.** *A. aegypti* larvae were treated with 100 ppm dose of these organic extracts separately. The percentage of larval mortality was calculated against the sham treated controls and represented as the mean ± SD of triplicates. Abbreviations: **Ace**, Acetone; **Eth**, Ethanol; **Hex**, Hexane; **Wat**, Water.
